# Inhibition of ERK5 activity ameliorates osteoarthritis by suppressing NLRP3 inflammasome-mediated chondrocyte pyroptosis

**DOI:** 10.1038/s41413-026-00573-x

**Published:** 2026-09-02

**Authors:** Baolin Zhang, Peihang Fang, Jiahui Long, Deying Su, Hengyu Liu, Ricong Weng, Gengjia Chen, Yingtong Chen, GuanHong Chen, Zhiheng Liao, Taifeng Zhou, Shuqing Chen, Peiqiang Su, Caixia Xu

**Affiliations:** 1 Department of Spine Surgery, The First Affiliated Hospital of Sun Yat-sen University, Guangzhou, China; 2 Guangdong Provincial Key Laboratory of Orthopedics and Traumatology, Department of Spine Surgery, The First Affiliated Hospital of Sun Yat-sen University, Guangzhou, China; 3 Research Center for Translational Medicine, First Affiliated Hospital, Sun Yat-sen University, Guangzhou, China; 4 Department of Traditional Chinese Medicine, the First Affiliated Hospital, Sun Yat-sen University, Guangzhou, China

**Keywords:** Diseases, Bone

## Abstract

Osteoarthritis (OA) is a widespread degenerative joint disorder manifesting as chronic pain, functional impairment, and progressive disability, imposing a substantial burden on global public health. Despite extensive research, the precise molecular pathways underlying OA pathogenesis remain incompletely elucidated. Our data indicated that human OA cartilage and cartilaginous tissues from aged mice exhibited markedly elevated levels of extracellular signal-related kinase 5 (ERK5) enzyme activity. Using *Col2a1-CreER*^*T2*^/*Erk5*^flox/flox^ mice, we found that *Erk5* deletion in cartilage inhibited the progression of post-traumatic and aging OA mouse while constitutive activation of ERK5 significantly accelerated OA development via intra-articular injection of adeno-associated virus model. Mechanistically, ERK5 interacted with the PYD domain of NLRP3 and mediated NLRP3 phosphorylation at serine 198, which facilitated mature inflammasome assembly, triggered pyroptosis and subsequently exacerbated OA progression. Furthermore, we identified a small compound, Oroxylin A, which attenuated ERK5 activation and effectively ameliorated the development of post-traumatic and aging-induced OA in mice. Taken together, our studies demonstrate that ERK5 is a key regulator effecting chondrocytes inflammation and pyroptosis through activating NLRP3 inflammasome and ERK5 is a potential therapeutic target for OA treatment.

## Introduction

Osteoarthritis (OA) is a degenerative joint disease characterized by persistent pain, joint swelling, and restricted articular movement.^1,2^ Its pathological features include damage to articular cartilage, subchondral bone remodeling, synovitis, and osteophyte formation.^3,4^ The OA prevalence is progressively rising as a result of demographic aging and the worldwide obesity crisis, leading to substantial societal burdens and representing a critical challenge to public health.^5,6^ At present, the management of OA focues on alleviating symptoms, while joint replacement surgery serves as a final option for more severe cases.^7,8^ And no disease-modifying drugs for osteoarthritis (DMOADs) have been developed.^9^ Although several potent drugs are undergoing various phases of clinical trials, none can halt or reverse OA progression, likely due to its high heterogeneity at both pathological and molecular levels. Therefore, elucidating the underlying molecular pathogenesis of OA is essential for the development of effective therapeutic interventions.

Extracellular signal-related kinase 5 (ERK5), a serine/threonine kinase within the mitogen-activated protein kinases (MAPKs) superfamily, is involved in transducing a wide array of stimuli such as mitogens, inflammatory cytokines, oxidative and osmotic stress, mechanical stimuli, and others.^10–17^ These signals can induce distinct cellular responses. Studies have demonstrated that ERK5 may act as a negative regulator during early chondrocyte differentiation.^18^ Inhibition of ERK5 promotes increased expression of SOX9 and attenuates chondrocyte hypertrophic differentiation.^19,20^ In addition, ERK5 serves as a critical regulator of inflammation responses.^21–24^ Previous studies illustrated that blocking ERK5/MEK5 signaling reduces inflammation induced by pathogens and cytokines (IL-1β, TNF-α), protecting against sepsis and lung ischemia-reperfusion injury in mice.^21^ The ERK5 inhibitor XMD8-92 attenuated retinal oxidative stress and inflammatory responses, thereby inhibiting vascular hyperpermeability and capillary degeneration in diabetic murine retinas.^25^ Those studies indicate that ERK5 is a potential therapeutic target in diverse inflammatory diseases. As mentioned above, ERK5 promotes chondrocyte hypertrophy and mediates inflammatory responses, processes closely linked to OA pathogenesis. We speculate that ERK5 may participate in OA progression.

To define the essential roles and uncover the molecular mechanisms of ERK5 in chondrocyte-mediated OA regulation, we implemented a comprehensive investigative strategy combining both in vivo and in vitro models. Consequently, we identified ERK5 as a promising target for therapeutic intervention in OA.

## Results

### Elevated p-ERK5 levels in OA cartilages of human and mice

**Fig. 1:**
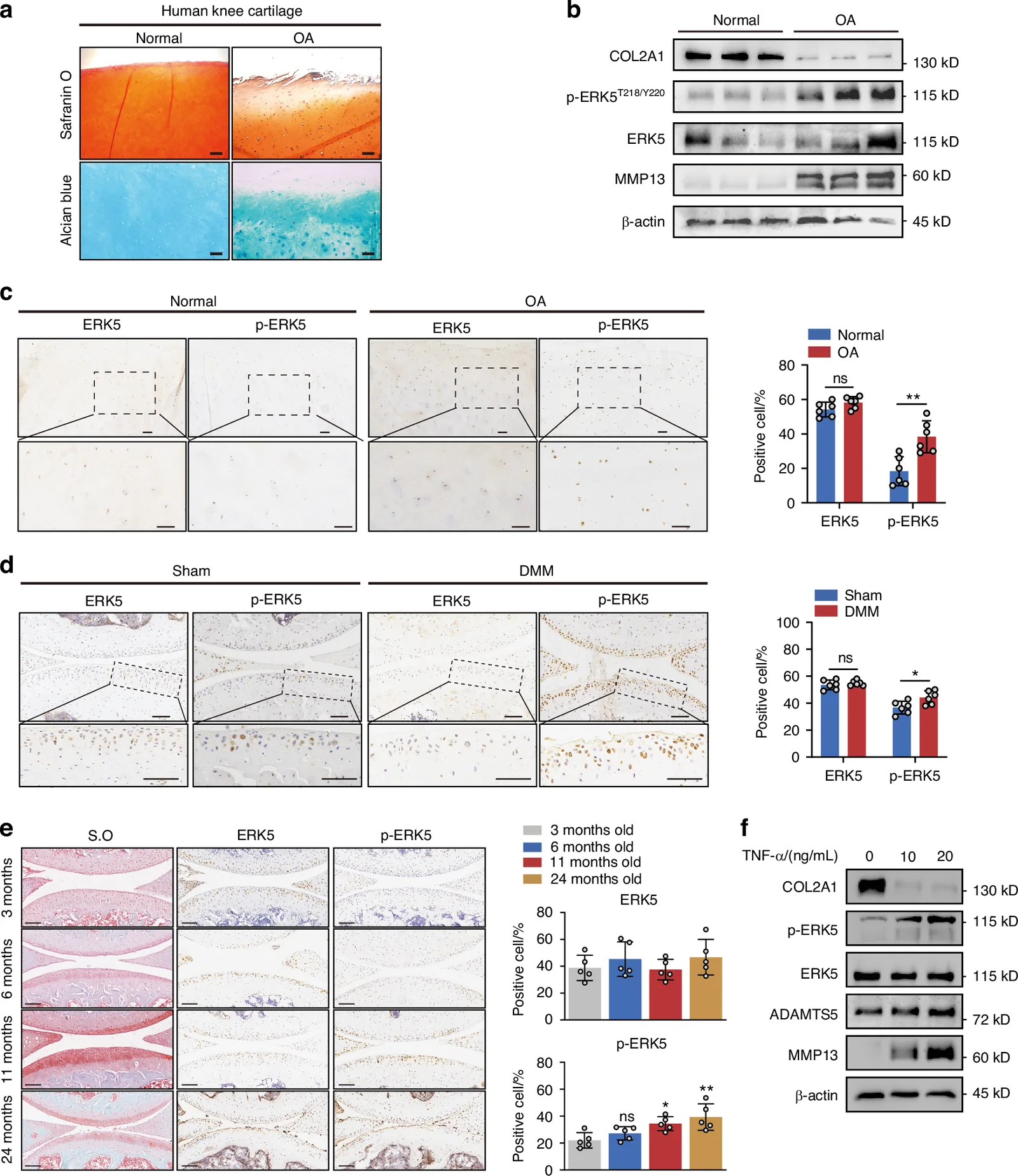
Elevated p-ERK5 levels in human and mice OA cartilage. **a** Images of safranin O/fast green staining and Alcian blue staining of human cartilages. Scale bars, 100 μm. **b** Immunoblotting of COL2A1, p-ERK5^T218/Y220^, ERK5, MMP13 and β-Actin in human cartilage tissues. Three independent biological replicates per group. **c** Immunohistochemistry results of ERK5 and p-ERK5^T218/Y220^ of human cartilages from OA or control group (*n* = 6). Scale bars, 100 μm. **d** Representative images of immunohistochemistry of ERK5 and p-ERK5^T218/Y220^ in knee joint cartilages from sham and DMM mice at 8 weeks after surgery (*n* = 6). Scale bars, 100 μm. **e** Results of safranin O/fast green staining and immunohistochemistry of ERK5 and p-ERK5^T218/Y220^ in knee joint cartilages of wild-type mice at various ages (*n* = 6). Scale bars, 100 μm. **f** Immunoblotting results in primary mouse chondrocytes treated with TNF-α (10 ng/mL or 20 ng/mL) for 48 h. Two-tailed unpaired *t* test is used for (**c**, **d**). One-way ANOVA followed by Tukey’s multiple comparisons is used for (**e**). Data are presented as the mean ± SD; ns (non-significant), *P < 0.05, **P < 0.01

To determine the role of ERK5 in OA, we first collected the cartilage specimens from OA patients and controls. OA cartilage showed greater cartilage damage and a thinner cartilage layer compared to normal controls, as indicated by S.O and Alcian blue staining (Fig. 1a). Western blot analysis indicated a marked elevation of phospho-ERK5 (p-ERK5) protein levels in damaged cartilages. Immunohistochemistry staining furtherly confirmed the results (Fig. 1b, c). Following the induction of post-traumatic OA via DMM surgery, a significant increase in p-ERK5 levels was observed compared to sham controls (Fig. 1d). As OA is fundamentally associated with aging, we investigated ERK5 and p-ERK5 expression in the articular cartilage of wild-type mice at various ages. The results showed that p-ERK5 exhibited a progressive elevation with advancing age (Fig. 1e). In addition, we used TNF-α-stimulated chondrocytes to establish an in vitro OA model. Similarly, the expression of p-ERK5 increased under inflammation condition (Fig. 1f). These findings implied that p-ERK5 levels were markedly elevated in both humans and mouse OA cartilage.

### Ablation of ERK5 in adult cartilage alleviates the development post-traumatic and aged-related OA in mice

**Fig. 2:**
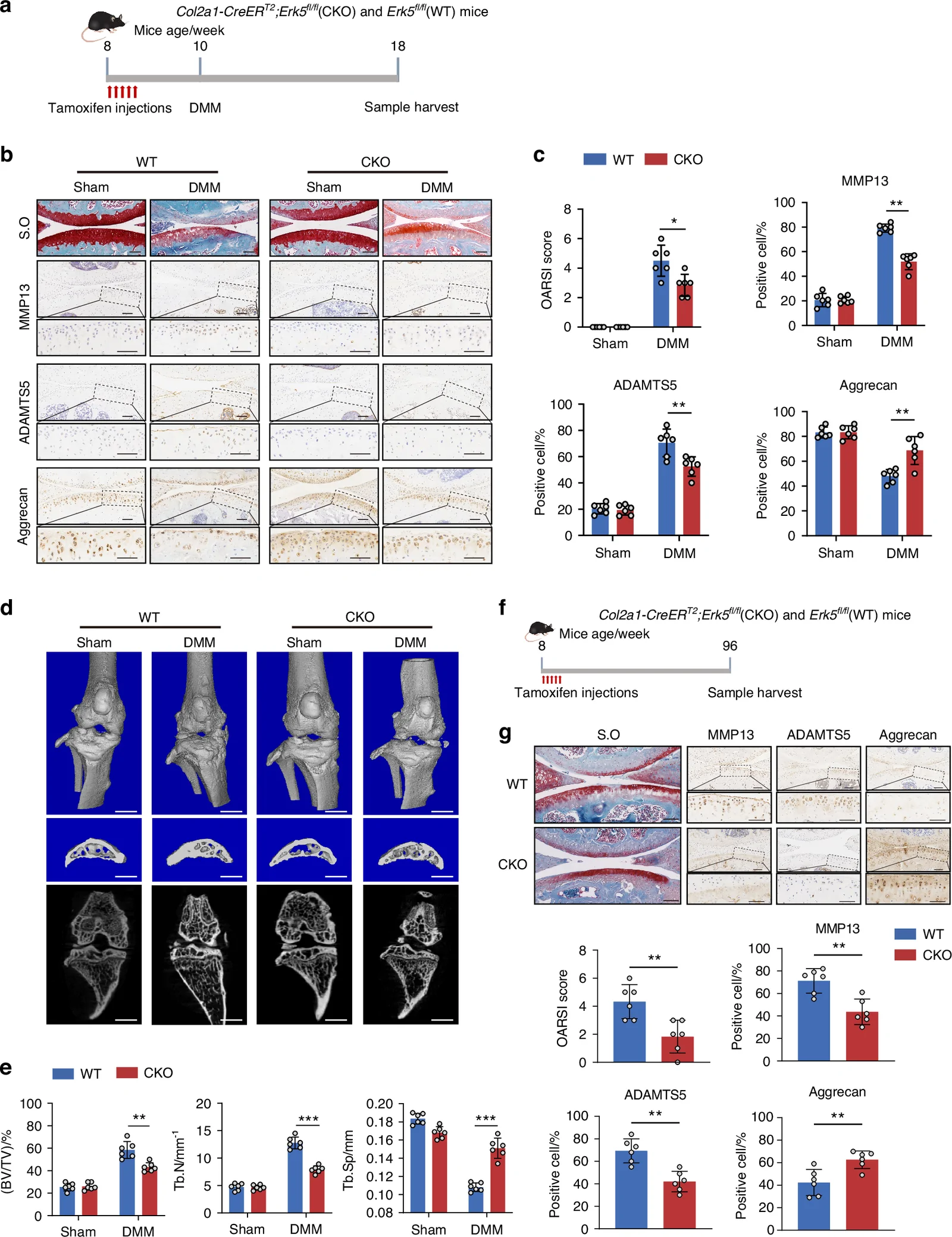
Ablation of ERK5 in adult cartilage alleviates the progression of post-traumatic and aged-related OA in mice. **a** Experimental scheme. Eight-week-old CKO and WT mice received intraperitoneal injections of tamoxifen once daily for five consecutive days. Two weeks after injection, all mice were subjected to sham or DMM surgery. Knee joints were then collected 8 weeks later. **b** Images of safranin O/fast green staining and immunohistochemistry of MMP13, ADAMTS5 and Aggrecan in knee joint cartilages of mice after sham or DMM surgery. Scale bars, 100 μm. **c** The OARSI grades and quantification of immunohistochemical results are shown (*n* = 6). **d** Representative images of micro-CT scanning of knee joint of WT and CKO mice at 8 weeks after sham or DMM surgery. **e** Quantification of the BV/TV, Tb.N and Tb.Sp by static histomorphometry. **f** Experimental scheme. 8-week-old CKO and WT mice were administrated with five daily injections of tamoxifen. Knee joints were harvested at 96 weeks of age. **g** Representative images of safranin O/fast green staining and immunohistochemistry of MMP13, ADAMTS5 and Aggrecan in knee joint cartilages of WT and CKO mice at 96 weeks old (*n* = 6). Two-way ANOVA test followed by Tukey’s post hoc analysis is used for immunohistochemistry and micro-CT analysis in (**c**, **e**). Two-tailed unpaired *t* test is used for immunohistochemistry analysis in (**g**). Mann–Whitney U test is used for OARSI scores in (**c**, **g**). Data are presented as the mean ± SD; ns (non-significant), *P < 0.05, **P < 0.01, ***P < 0.001

To investigate the function of ERK5 in OA pathogenesis, we first assessed the effect of ERK5 knockdown on cartilage extracellular matrix (ECM) degradation in vitro. The efficiency of the ERK5 knockdown was confirmed via WB results (Fig. S1A). Under basal conditions, ERK5 knockdown did not affect COL2A1 (ECM anabolic markers), MMP13 or ADAMTS5 (ECM catabolic markers) expression. Upon TNF-α stimulation, ERK5 knockdown significantly reduced MMP13 and ADAMTS5, but did not alter COL2A1 levels (Fig. S1B). Furthermore, the role of ERK5 in OA was examined using inducible conditional knockout mice (CKO, *Col2a1-CreER*^*T2*^; *Erk5*^flox/flox^), because *Erk5* early deficiency impaired articular cartilage development.^26^ We first validated the knockout efficiency using genotyping, qRT-PCR, and immunohistochemistry (Fig. S2A-D). To determine the effect of ERK5 genetic ablation on cartilage degeneration, we compared disease severity in *Erk5*^flox/flox^ (WT) and CKO mice following DMM surgery (Fig. 2a). Safranin O staining demonstrated comparable knee joint histomorphometry between WT and CKO mice before DMM surgery. However, WT mice exhibited significantly reduced cartilage thickness and larger deterioration areas relative to CKO mice after surgery. OARSI scores were lower in CKO mice than in WT groups. The immunohistochemical staining assay revealed that decreased expression of Aggrecan in cartilage of WT mice compared with CKO mice and ERK5 deficiency also significantly reduced levels of MMP13 and ADAMTS5 (Fig. 2b, c). Additionally, micro-CT analysis of tibial subchondral bone revealed no structural differences between WT and CKO mice prior to surgery. However, following DMM surgery, CKO mice displayed a marked reduction in both osteophyte formation and subchondral bone thickening compared with WT mice (Fig. 2d, e). Moreover, we evaluated the association of ERK5 with OA pain using open field experiment and von Frey testing. Following DMM surgery, CKO mice exhibited increased total movement distance and a significant reduction in mechanical allodynia over the 12-week observation period (Fig. S3A, B). We investigated whether adult cartilage-specific ERK5 deletion attenuated age-related OA development (Fig. 2f). Aged (96-week-old) CKO mice exhibited reduced MMP13 and ADAMTS5 levels and increased Aggrecan expression compared to age-matched controls. Moreover, *Erk5* knockout ameliorated spontaneous cartilage destruction according to OARSI scores (Fig. 2g). Collectively, those results indicated that cartilage-specific *Erk5* deletion in knee joints inhibited the development of post-traumatic and aging-related OA.

### Constitutive activation of ERK5 enhance osteoarthritis in mice

**Fig. 3:**
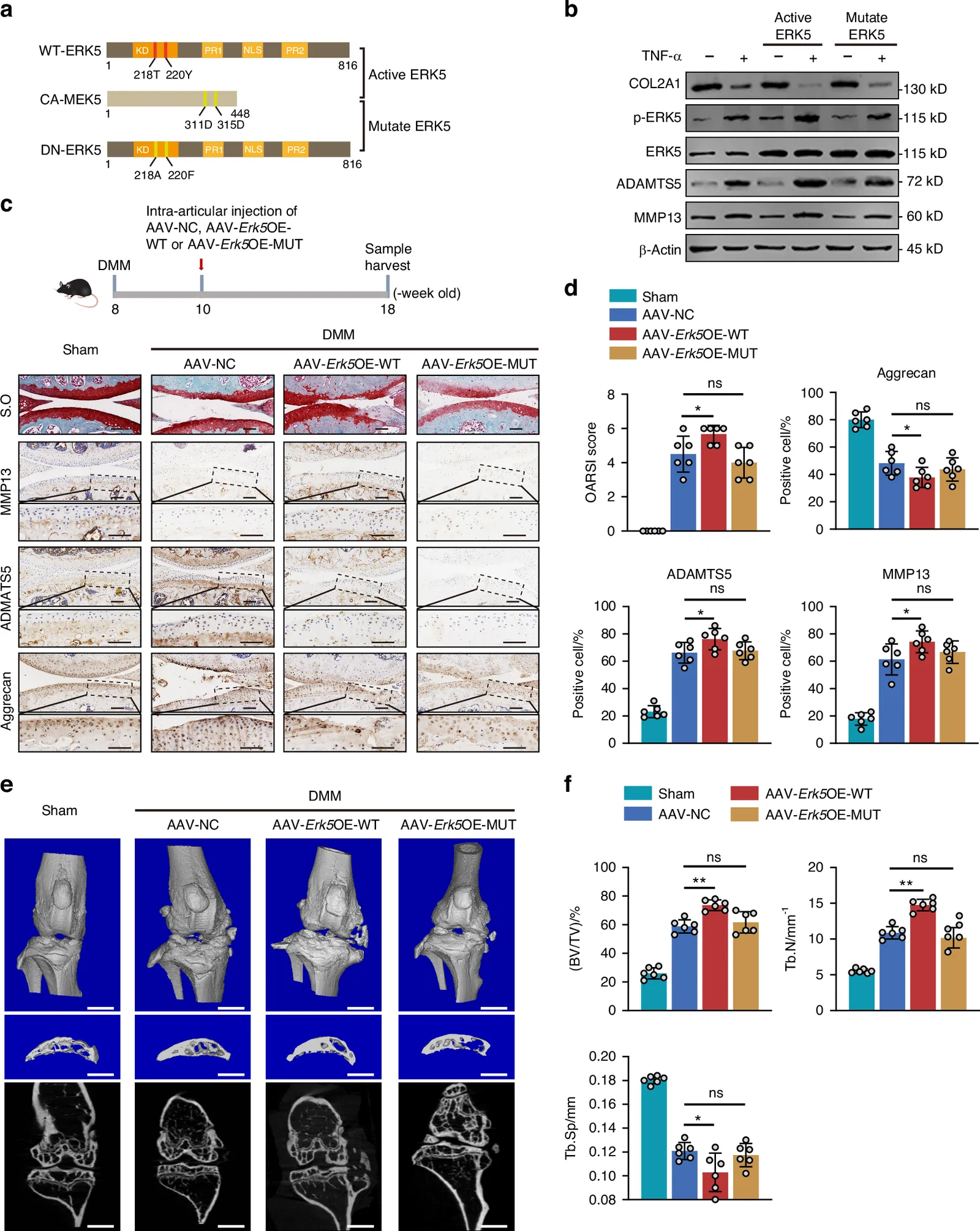
Constitutive activation of ERK5 promotes OA progression in mice. **a** Schematic illustration of the WT-ERK5, CA-MEK5 and the DN-ERK5. **b** Immunoblotting of indicated proteins in chondrocytes with different plasmids transfection. **c** Images of safranin O/fast green staining and immunohistochemistry of MMP13, ADAMTS5 and Aggrecan in knee joint cartilages from Sham, DMM + AAV-NC, DMM + AAV-*Erk5*OE-WT, and DMM + AAV*-Erk5*OE-MU groups. Scale bars, 100 μm. **d** The OARSI grades and quantification of immunohistochemical results are shown (*n* = 6). **e** Images of micro-CT scanning of knee joint from Sham, DMM + AAV-NC, DMM + AAV-*Erk5*OE-WT, and DMM + AAV*-Erk5*OE-MU groups. **f** Quantitative analysis of results from (**e**) (*n* = 6). One-way ANOVA followed by Tukey’s multiple comparisons is used for immunohistochemistry and micro-CT analysis in (**d**, **f**). Kruskal–Wallis test is used for OARSI scores in (**d**). Data are presented as the mean ± SD; ns (non-significant), *P < 0.05, **P < 0.01, ***P < 0.001

Next, we explored the effect of constitutive ERK5 activation in OA pathogenesis. First of all, we modulated ERK5 activity in chondrocytes via transiently transfecting with different plasmids. Cells were co-transfected with constitutively active MEK5 (CA-MEK5) plasmid alongside either wild-type ERK5 (WT-ERK5) or dominant-negative ERK5 (DN-ERK5) incapable of phosphorylation by MEK5 (Fig. 3a). The result showed that persistent ERK5 activation by MEK5 upregulated MMP13 and ADAMTS5 in an in vitro OA model. But co-expression of CA-MEK5 and DN-ERK5 blocked those effects in chondrocytes (Fig. 3b).

To validate the ERK5 activation effect in chondrocyte during OA cartilage destruction in vivo, we injected different dual AAV vectors into mouse knee joints (Fig. 3c). The adult mice were administered an intra-articular injection after DMM surgery, and joint samples were subsequently harvested 8 weeks later. The AAV infection was verified by assessing Green fluorescent protein (GFP) expression via IF analysis (Fig. S4A). And the ratio of p-ERK5 to total ERK5 was examined by immunofluorescence, further verifying successful ERK5 activation (Fig. S4B). Mice in *Erk5*OE-WT group had higher OARSI grades relative to the AAV-NC group, whereas *Erk5*OE-MUT group showed no significant increase. IHC analysis revealed that mice in the *Erk5*OE-WT group displayed reduced Aggrecan levels and elevated MMP3 and ADAMTS5 expression compared to the AAV-NC group, whereas the *Erk5*OE-MUT group exhibited no significant alterations (Fig. 3d). Micro-CT analysis showed that *Erk5*OE-WT group developed more severe OA-related pathologies like osteophyte formation and subchondral bone sclerosis (Fig. 3e, f). Totally, these results show that constitutive activation of ERK5 significantly accelerates OA progression in mice.

### ERK5 is associated with chondrocyte pyroptosis in OA

**Fig. 4:**
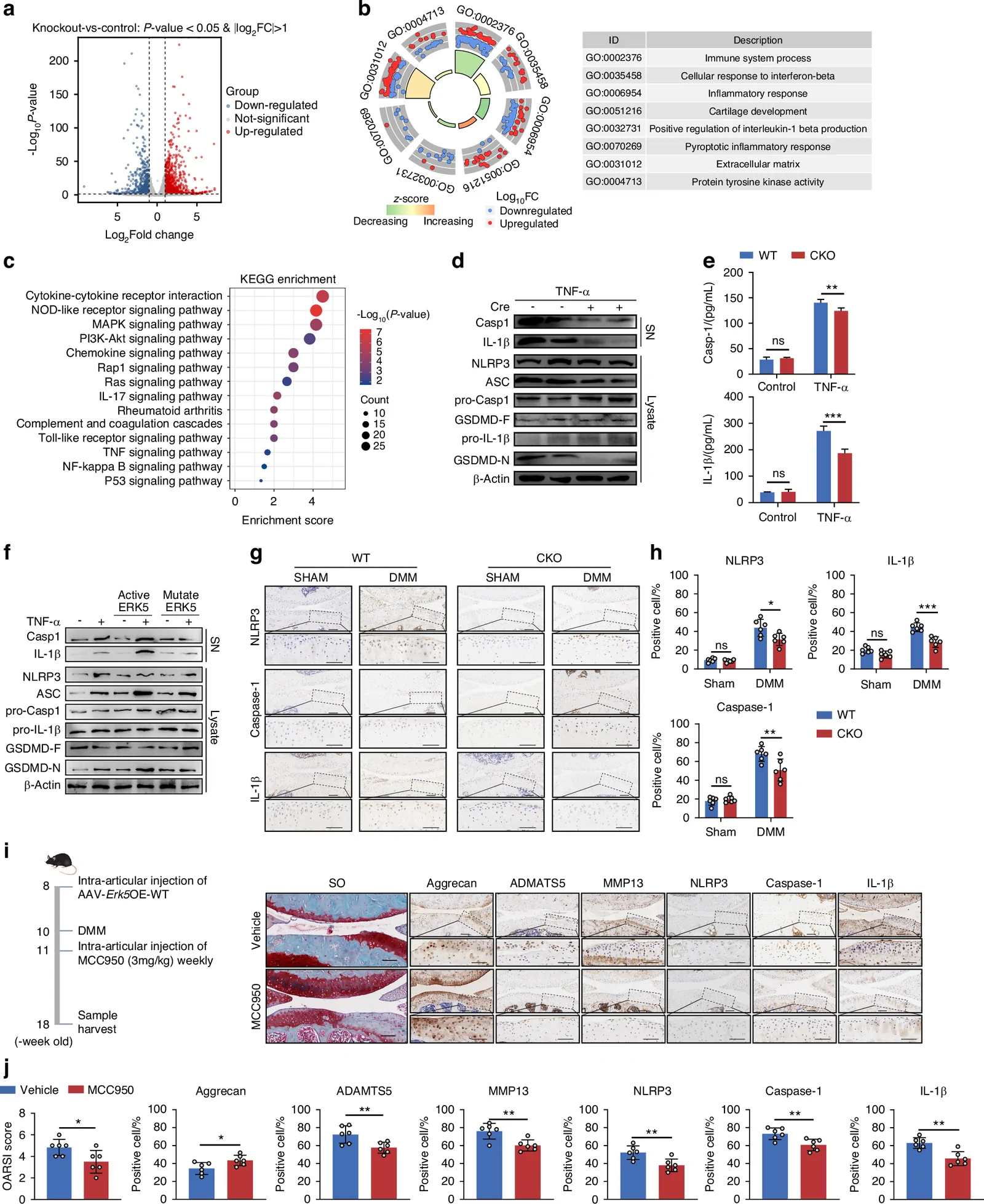
ERK5 is associated with chondrocyte pyroptosis in OA. **a** The results of RNA-seq volcano plot (|log_2_Fold change| > 1, FDR < 0.05). **b** Gene Ontology terms and pathways of upregulated and downregulated DEGs of RNA-seq data. **c** KEGG pathway enrichment for significantly downregulated genes (*P* < 0.05) from RNA-seq. **d** Immunoblotting results of primary articular chondrocytes treated with or without 4-OHT under TNF-α stimulation. **e** Measurement of IL-1β and Caspase-1 in chondrocyte supernatants by ELISA following TNF-α stimulation. **f** Immunoblotting of indicated proteins in chondrocytes with different plasmids transfection. **g** Images of immunohistochemistry results in knee joint cartilages of WT and CKO mice at 8 weeks after sham or DMM surgery. Scale bars, 100 μm. **h** Quantitative analysis of results from (**g**) (*n* = 6). **i** Images of immunohistochemistry of different markers in mouse joints following intra-articular AAV-*Erk5*OE-WT injection, with or without MCC950 treatment after DMM surgery. Scale bars, 100 μm. **j** Quantitative assessment of results from **i** (*n* = 6). Two-way ANOVA test followed by Tukey’s post hoc analysis is used for (**e**, **h**). Two-tailed unpaired *t* test is used for immunohistochemistry analysis in (**j**). Mann–Whitney U test is used for OARSI scores in (**j**). Data are presented as the mean ± SD; ns (non-significant), *P < 0.05, **P < 0.01, ***P < 0.001

To elucidate the molecular mechanism underlying the regulation of OA by ERK5, we performed RNA-seq analysis after knockout of *Erk5* in mouse chondrocytes (Fig. 4a). Gene Ontology (GO) enrichment analysis revealed enrichment of the pyroptotic inflammatory response (Fig. 4b). Kyoto Encyclo pedia of Genes and Genomes (KEGG) analysis demonstrated that inflammation-related signaling pathway was markedly enriched (Fig. 4c). Therefore, we speculated that ERK5 may regulate the development of OA by modulating pyroptosis. To investigate the potential function of ERK5 in regulating pyroptosis, we detected pyroptosis-related molecules change of downregulating ERK5 in the chondrocyte inflammation model. The results showed that ERK5 knockout reduced levels of IL-1β and caspase-1 (Fig. 4d). ELISA revealed significantly reduced secretion levels of caspase-1 and IL-1β in ERK5-deficient chondrocytes relative to controls (Fig. 4e). Similarly, utilizing an in vitro model with constitutive ERK5 activation, we observed that ERK5 activation elevated pyroptosis-related markers, whereas ERK5-Mut suppressed these effects in chondrocytes (Fig. 4f). Further, we explored the impact of ERK5 on pyroptosis in vivo, the results demonstrated that ERK5 knockout reduced the levels of cartilage pyroptosis markers (Fig. 4g, h). To substantiate the contribution of the NLRP3 inflammasome to the ERK5-mediated effect on OA, we assessed whether suppressing the NLRP3 inflammasome could reverse the detrimental effect of constitutive ERK5 activation on DMM mice. Compared with vehicle treatment, the administration of MCC950 (an NLRP3 inflammasome inhibitor) markedly reduced the levels of Caspase-1 and IL-1β in *Erk5*OE-WT mice. And MCC950 treatment ameliorated progression of osteoarthritis in *Erk5*OE-WT mice, with reduced MMP13 and ADAMTS5 levels (Fig. 4i-j). Therefore, inhibition of NLRP3 activation rescued constitutive ERK5 activation-induced osteoarthritis enhancement. In summary, ERK5 is essential for regulating chondrocyte pyroptosis in OA while blockade of the NLRP3 inflammasome counteracts the OA aggravation induced by persistent ERK5 activation.

### ERK5 co-localizes and interacts with NLRP3

**Fig. 5:**
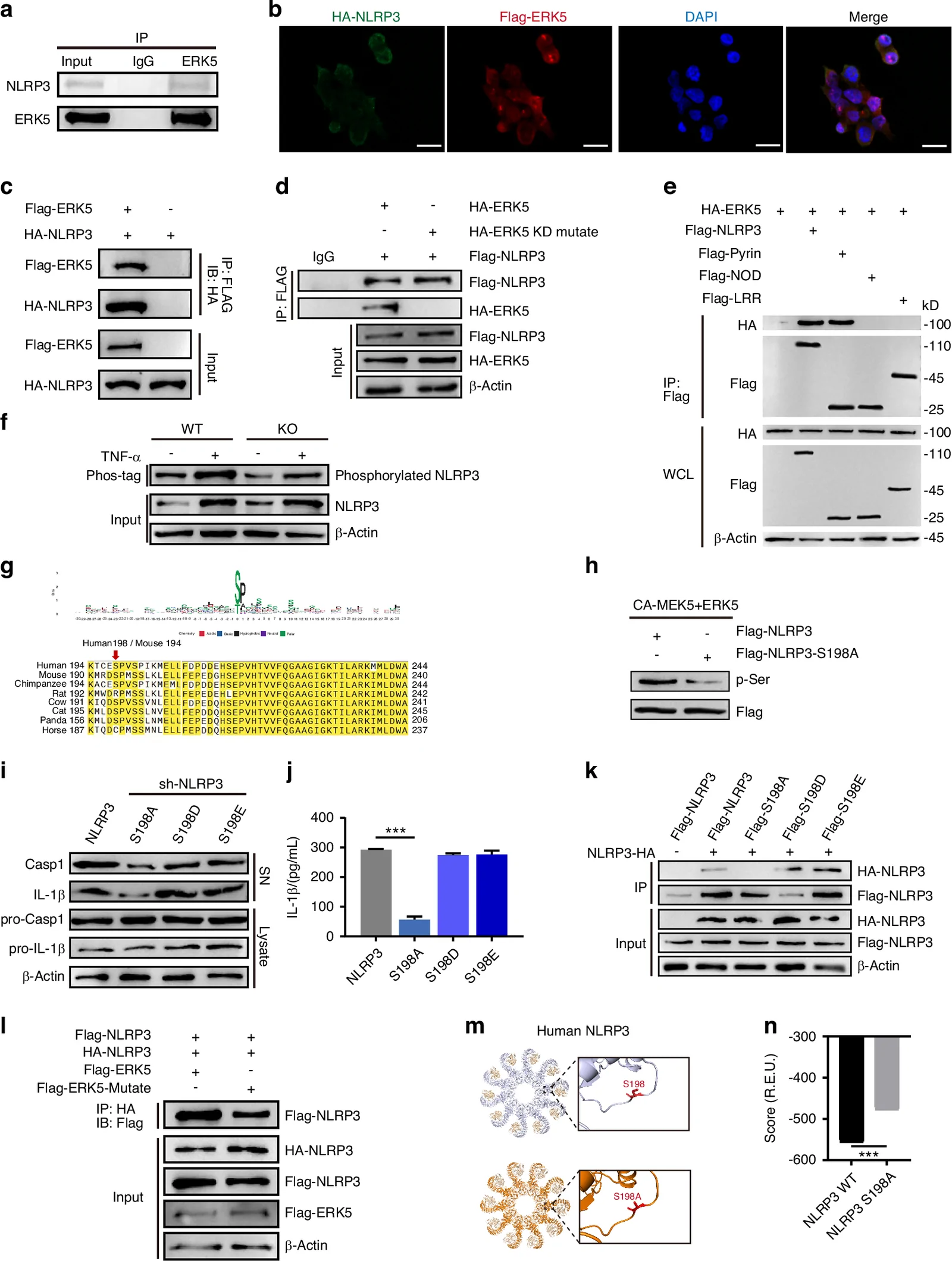
ERK5 phosphorylates NLRP3 at serine 198 and promotes inflammasome assembly. **a** Co-IP analysis of the NLRP3–ERK5 interaction in lysates from TNF-α-stimulated chondrocytes. **b** Confocal images of HEK293T cells overexpressing HA-NLRP3 (green) and Flag-ERK5 (red). Scale bar, 50 μm. **c** Co-IP analysis of Flag-ERK5 and HA-NLRP3 interaction. **d** Co-IP analysis of Flag-NLRP3 interaction with HA-ERK5 wild-type and T218A/Y220F mutant. **e** Co-IP analysis of HA-ERK5 and full/truncated NLRP3 interaction. **f** Phos-tag^TM^ SDS–PAGE results for phosphorylated NLRP3 levels in chondrocytes with or without TNF-α stimulation. **g** Prediction of ERK5 binding motif and alignment of NLRP3 sequences containing potential phosphorylation sites of different species. **h** The levels of serine phosphorylation of wild-type NLRP3 or NLRP3-S198A following incubation with active ERK5 via in vitro kinase assay. **i** Immunoblotting of indicated proteins from chondrocytes transduced with different lentivirus. **j** ELISA of IL-1β. **k** Co-IP and immunoblotting analysis of NLRP3 aggregation in HEK293T cells co-expressing HA-tagged NLRP3 with different Flag-tagged NLRP3 constructs. **l** Co-IP and immunoblotting analysis of NLRP3 aggregation in HEK293T cells co-expressing Flag-ERK5, Flag-ERK5^T218A/Y220F^, and different tagged NLRP3. **m** The structural model of WT NLRP3-NEK and S198A mutant (serine 198 is shown in red). **n** The structural stability score (expressed in R.E.U.) for the wild-type NLRP3-NEK and its S198A mutant was calculated by Rosetta. Two-tailed unpaired *t* test is used for (**n**). One-way ANOVA is used for (**j**). Data are presented as the mean ± SD; ns (non-significant), *P < 0.05, **P < 0.01, ***P < 0.001

Given that NLRP3 is pivotal in inflammasome activation and its phosphorylation represents a key post-translational modification mechanism governing this process, we hypothesized that ERK5 directly interacts with NLRP3 and mediates its phosphorylation. Immunoprecipitation (IP) assays of chondrocyte found that endogenous ERK5 kinase bound to NLRP3 (Fig. 5a). Immunofluorescence analysis demonstrated significant colocalization of ERK5 and NLRP3 (Fig. 5b). Additionally, when co-expressed in HEK293T cells, ERK5 kinase was observed to interact with NLRP3 (Fig. 5c). We further delineated the specific protein domains mediating the ERK5-NLRP3 interaction. Our findings revealed that the ERK5 kinase domain directly interacted with NLRP3, whereas the ERK5 kinase mutant^T218A/Y220F^ (abolished kinase activity) lost NLRP3-binding capacity (Fig. 5d). These data underscore the necessity of ERK5 enzymatic activity for successful engagement with the NLRP3 inflammasome.

To identify the key domains mediating the NLRP3-ERK5 interaction, we undertook a series of domain-mapping experiments. NLRP3 is composed of PYRIN (PYD), NACHT (nucleotide binding domain or NBD), and LRR domains. Co-IP results indicated that ERK5 associates exclusively with the PYD domain (Fig. 5e). Taken together, these data demonstrated that ERK5 drives inflammasome activation via direct interaction with the NLRP3 PYD domain.

### ERK5 phosphorylates NLRP3 at serine 198 and promotes inflammasome assembly

ERK5 belongs to the serine/threonine kinase family and participates in the phosphorylation of multiple proteins. Previous Co-IP results showed that ERK5 interacted with NLRP3 by binding to the PYD domain. Therefore, we hypothesized that ERK5 plays an essential role in regulating NLRP3 inflammasome activation by directly phosphorylating NLRP3. To test this hypothesis, we used Phos-tag^TM^ SDS–PAGE to detect the phosphorylation of NLRP3. Erk5 knockout chondrocytes showed a significant reduction in NLRP3 phosphorylation (Fig. 5f). To further illustrate the specific phosphorylation site of NLRP3, we used bioinformatics tools to analyze the binding sequence of ERK5 kinase. The results showed that the S198 site (S194 in mouse) is a potential phosphorylation site on NLRP3 targeted by ERK5, which is mostly conserved across different species (Fig. 5g). To validate S198 as the direct target of ERK5, we performed an in vitro kinase assay by incubating active ERK5 with either recombinant wild-type NLRP3 or NLRP3^S198A^. We observed that serine phosphorylation was significantly attenuated in NLRP3^S198A^ compared with wild-type NLRP3, indicating that ERK5 directly phosphorylates NLRP3 on S198 (Fig. 5h). To find out how the phosphorylation of S198 influenced NLRP3 activation, we overexpressed WT NLRP3 and different NLRP3 mutants in NLRP3 knockdown chondrocytes. The results indicated that NLRP3^S198D^ and NLRP3^S198E^ (to mimic phosphorylation) restored IL-1β and caspase-1 levels, but NLRP3^S198A^ did not trigger these effects (Fig. 5i, j). Totally, those results suggest that phosphorylation at Ser198 by ERK5 is crucial for activating NLRP3.

Given that NLRP3 inflammasome assembly is vital for activating NLRP3, we explored the influence of ERK5-mediated S198 phosphorylation of NLRP3 on inflammasome assembly. Co-transfection assay of different Flag-NLRP3 structures with HA-NLRP3 revealed that S198A mutation restricted NLRP3 self-interaction while the S198D and S198E mutations exhibited enhanced interaction (Fig. 5k). Additionally, transfection with a kinase-inactive ERK5 mutant suppressed NLRP3 self-interaction (Fig. 5l). We conducted symmetric docking on the human NLRP3-NEK7 complex to investigate potential phosphorylation-induced conformational changes, which was conserved in different organisms (Fig. 5m). NLRP3^S198A^ mutant markedly decreased the complex’s docking score relative to the wild-type. In other words, the S198A mutation destabilized the structure of the NLRP3-NEK7 complex (Fig. 5n). These results revealed NLRP3 S198 phosphorylation is critical for facilitating NLRP3 interactions. Taken together, these data demonstrated that phosphorylation of NLRP3 at Ser198 by ERK5 promotes NLRP3 activation and inflammasome assembly.

### Oroxylin A bound to ERK5 and inhibited its Activity

**Fig. 6:**
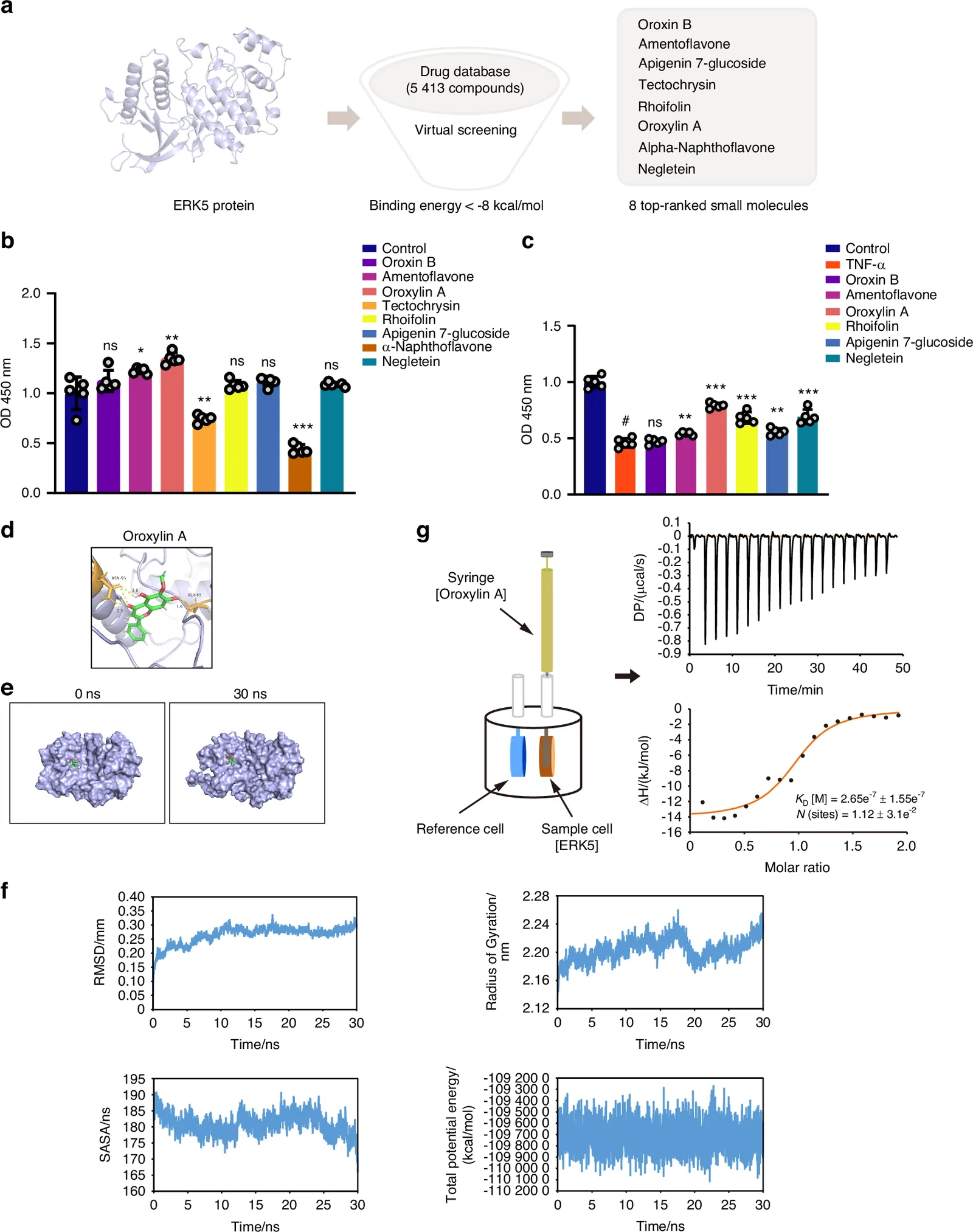
Oroxylin A bound to ERK5. **a** Using virtual screening to identify the potential compounds bound to human ERK5. **b** The cell proliferation rate was evaluated via a CCK8 assay following the treatment with various compounds. **c** The viability of mouse chondrocytes was assessed following treatment with various compounds under TNF-α stimulation. **d** The structure of Oroxylin A-ERK5 complex. **e** Surface display of the ERK5-Oroxylin A complex structure at 0 and 30 ns. **f** Plots of root mean square deviation (RMSD), radius of gyration, solvent accessible surface Area (SASA) and potential energy profiles of during the molecular dynamics simulation. **g** The binding affinity (*K*_D_) and stoichiometry (N) were determined by isothermal titration calorimetry (ITC) at 25 °C. Thermogram fitting was conducted using the MicroCal PEAQ-ITC software using default parameters. One-way ANOVA followed by Tukey’s multiple comparisons is used for (**b**, **c**). Data are *P* resented as the mean ± SD; ns (non-significant), *P < 0.05, **P < 0.01, ***P < 0.001

To identify pharmacological agents capable of targeting ERK5 for OA therapy, we conducted molecular docking screen of natural small molecules. Candidates were prioritized based on high binding affinity and relevant biological activities (e.g., anti-inflammatory or anti-oxidative properties), yielding eight potential compounds (Fig. 6a). Specifically, six compounds, including Oroxin B, Amenthoflavone, Oroxylin A, Apigenin 7-glucoside, Rhoifolin and Negletein had no adverse impact on chondrocyte viability, based on CCK-8 assay (Fig. 6b), and six compounds did not influence *ERK5* transcription (Fig. S5A). We next explored the effects of these small-molecule drugs on chondrocyte viability under inflammatory stimulation. The results showed that Oroxylin A exhibited the most pronounced effect in improving cell viability (Fig. 6c). Therefore, we chose Oroxylin A for further study. And Oroxylin A treatment reduced the level of p-ERK5 in chondrocytes (Fig. S5B). Then we applied molecular dynamics (MD) simulation to assess the binding affinity and stability of the molecular complex. The three-dimensional binding conformation of the ERK5-Oroxylin A complex is presented in Fig. 6d. Oroxylin A remained stably situated at the center of the ERK5 binding pocket throughout the entire MD simulation (Fig. 6e). No break in the RMSD curve suggested conformational stability of the complex. Additionally, the Solvent-Accessible Surface Area (SASA) of the complex decreased over the course of the simulation and the complex exhibited stable rotational radii, suggesting that compound binding did not compromise ERK5 stability. These findings indicated that Oroxylin A formed a stable interaction with ERK5 (Fig. 6f). To further investigate the interaction between Oroxylin A and ERK5, we performed isothermal titration calorimetry (ITC). The assay revealed a dissociation constant (*K*_D_) of 26.5 μmol/L, indicating direct binding between the two molecules (Fig. 6g). Oroxylin A inhibited the upregulation of NLRP3 phosphorylation and suppressed the elevation of pyroptosis-related markers under inflammatory condition (Fig. S5C, D). Furthermore, we investigated Oroxylin A’s effect on protein binding between ERK5 and NLRP3 by Co-IP. The results showed that Oroxylin A had no significant impact on the direct binding between ERK5 and NLRP3 (Fig. S5E). However, Oroxylin A suppressed the elevated NLRP3 phosphorylation induced by ERK5 activation (Fig. S5F). These results indicated that Oroxylin A may modulate ERK5 activity rather than affecting its binding to protein targets.

### Oroxylin A treatment ameliorated mouse OA

**Fig. 7:**
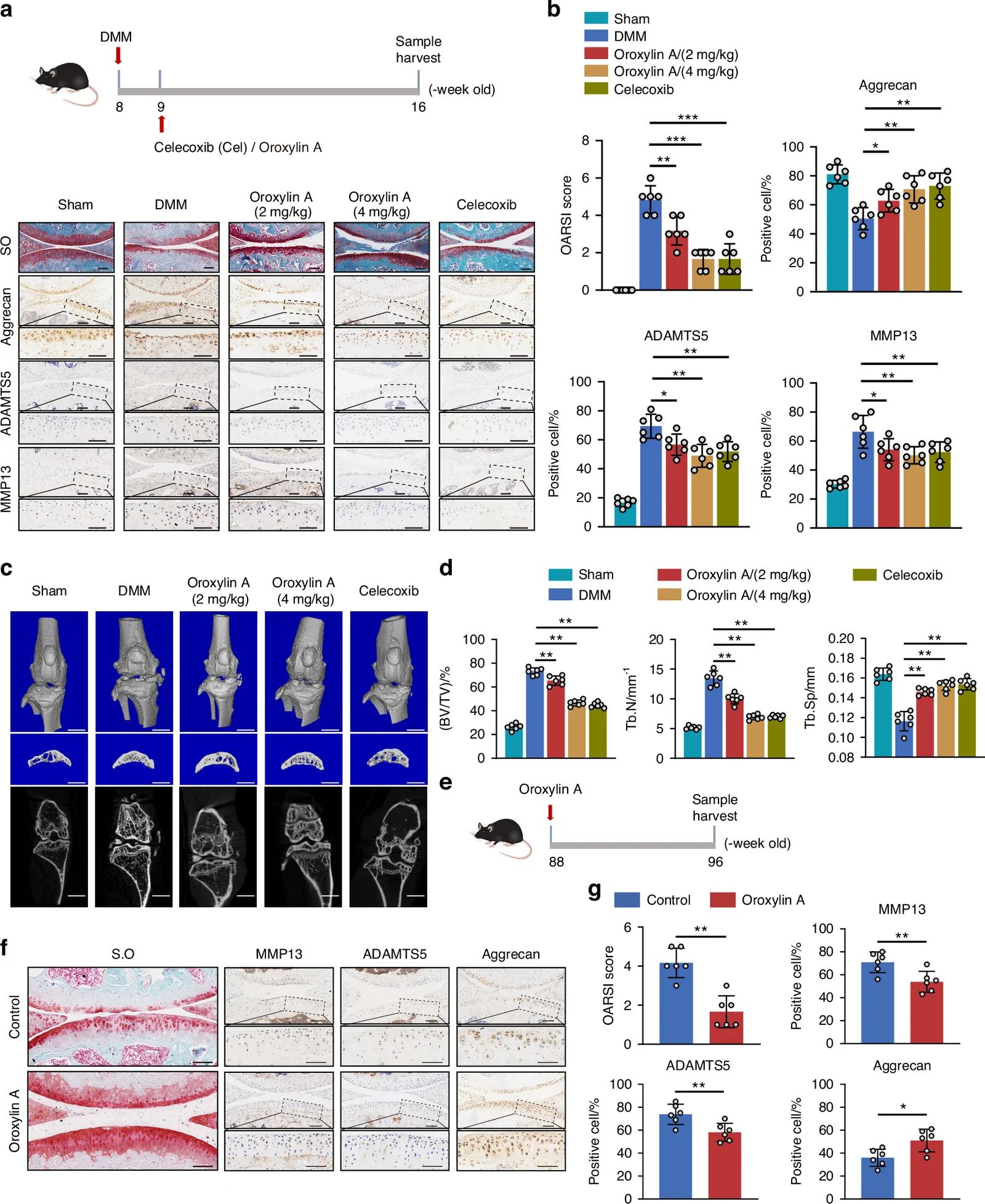
Oroxylin A treatment ameliorates mouse OA. **a** Images of safranin O/fast green staining and immunohistochemistry of MMP13, ADAMTS5 and Aggrecan in knee joint cartilages from sham, DMM, DMM+Oroxylin A (2 mg/kg), DMM+Oroxylin A (4 mg/kg) and celecoxib (20 mg/kg). Scale bars, 100 μm. **b** The OARSI grades and quantification of immunohistochemistry results are shown (*n* = 6). **c** Results of micro-CT scanning of knee joint from different groups. **d** Quantitative analysis of results from (**c**) (*n* = 6). **e** Experimental scheme of Oroxylin A Administration procedure in aged mice. **f** Images of safranin O/fast green staining and immunohistochemistry for MMP13, ADAMTS5, and Aggrecan in knee joint cartilages from differently aged mice with distinct treatments. **g** Quantitative analysis of results from (**f**) (*n* = 6). One-way ANOVA followed by Tukey’s multiple comparisons is used for immunohistochemistry analysis in (**b**) and micro-CT analysis in (**d**). Kruskal–Wallis test is used for OARSI scores in (**b**). Two-tailed unpaired *t* test is used for immunohistochemistry analysis in (**g**). Mann–Whitney U test is used for OARSI scores in (**g**). Data are presented as the mean ± SD; ns (non-significant), *P < 0.05, **P < 0.01, ***P < 0.001

Next, we investigated the effects of Oroxylin A on chondrocyte proliferation under inflammatory conditions. EdU staining revealed that TNF-α treatment suppressed DNA replication in chondrocytes, whereas Oroxylin A mitigated these inhibitory effects (Fig. S5G). Furthermore, we assessed the effect of Oroxylin A on TNF-α induced ECM degradation. The results indicated that Oroxylin A mitigated inflammatory effects by increasing COL2A1 expression and reducing ADAMTS5 and MMP13 levels (Fig. S6A). Alcian blue staining revealed that Oroxylin A rescued the TNF-α-induced loss of proteoglycans (Fig. S6B). Immunofluorescence staining further confirmed that Oroxylin A counteracted TNF-α-induced ECM degradation by upregulating COL2A1 and downregulating MMP13 (Fig. S6C, D). These results demonstrated that Oroxylin A effectively prevented chondrocyte degeneration in vitro. To assess the in vivo effect of Oroxylin A on OA progression in mice, we performed intra-articular injection of Oroxylin A at 2 mg/kg or 4 mg/kg doses per week while the positive control group was administered oral celecoxib (20 mg/kg) once daily. Mice treated with Oroxylin A exhibited less severe cartilage damage than those in the DMM group, as evidenced by Safranin O fast green staining and OARSI scores. IHC results indicated that compared to DMM group, Oroxylin A significantly upregulated the expression of Aggrecan while downregulating the expression of MMP3 and ADAMTS5 (Fig. 7a, b). Micro-CT results indicated that Oroxylin A significantly suppressed DMM-induced osteophyte formation and articular cartilage degradation (Fig. 7c, d). Additionally, mice in the Oroxylin A group had higher pain thresholds and increased movement distance compared to control group (Fig. S7A–C). Similar findings were observed in mouse aging model (Fig. 7e–g). In summary, those results demonstrated that Oroxylin A effectively ameliorates the development of DMM and aging-related OA in mice.

## Discussion

Cartilage degradation and disrupted homeostasis are characteristic features of OA, and arresting the progressive cartilage deterioration represents a promising therapeutic strategy.^6,27,28^ In this study, we investigated the function of ERK5 in OA. The level of p-ERK5 (enzymatically active form of ERK5) markedly upregulated in both the human and mouse OA cartilages. ERK5 deficiency in mice prevents the development of OA while constitutive activation of ERK5 promotes OA progression. Mechanistically, we found that ERK5 is associated with chondrocyte pyroptosis. At the molecular level, ERK5 physically interacts with the PYD domain of NLRP3 and phosphorylates NLRP3 at S198, thereby promoting the inflammasome assembly and induction of pyroptosis and accelerating OA. Furthermore, we identify a novel ERK5 inhibitor Oroxylin A via natural small compounds screening. In vivo experiment suggested that Oroxylin A alleviated cartilage degradation in OA mice, highlighting its potential clinical utility.

ERK5 plays an essential role in multiple cell signaling pathways and inflammation through phosphorylation cascades. Previous study revealed that SHP2 in neutrophils drove psoriasis by promoting NET formation via the ERK5 pathway. This process amplified skin inflammation through pro-inflammatory cytokine release.^24^ Seidita et al. reported that sphingosine-1-phosphate (S1P) activated the ERK5 pathway via the S1P1/3 receptor-SFK/MEK5 axis. This signaling cascade promoted reactive oxygen species production and pro-inflammatory factor expression in endometrial stromal cells, thereby driving the development of endometriosis.^23^ Wang et al. demonstrated that resveratrol (RSV) alleviated LPS-induced lung inflammation by inhibiting ERK5 nuclear translocation and activation. This effect modulated inflammatory responses through downregulation of pro-inflammatory mediators (IL-1β, IL-6, iNOS, COX-2) and upregulation of anti-inflammatory cytokine IL-10.^29^ In post-traumatic and aging-associated OA, we showed that genetic deletion of *Erk5* in cartilage ameliorated OA phenotypes based on histological and radiological assessments. Specifically, ERK5 loss alleviated inflammatory pain during OA progression. Previous evidence showed that peripheral inflammation activated the ERK5 pathway in DRG TrkA^+^ neurons. This activation upregulated TRPV1/TRPA1 expression, leading to thermal and cold hyperalgesia.^30^ Targeting sensory neuronal ERK5 may thus be a novel strategy against inflammatory pain, supporting its potential role in managing OA inflammatory pain. In addition, given the ERK5 is enzymatic active, future studies can utilize ERK5 mutant mice to further validate whether inhibiting ERK5 activation alleviates OA progression.

Chondrocyte pyroptosis driven by the NLRP3 inflammasome constitutes a critical cell death pathway in OA development.^31–33^ Upon activation, it recruits and activates caspase-1. Active caspase-1 cleaves GSDMD to create cell membrane pores, thereby initiating pyroptosis. It also cleaves pro-IL-1β into its mature form for secretion.^34,35^ In OA animal models, inhibiting NLRP3 inflammasome activation markedly reduced levels of pyroptosis-related proteins, subsequently alleviating synovitis and pain symptoms.^36,37^ Bai et al. showed that activation of the adenosine A3 receptor attenuated osteoarthritis development and pain by suppressing the ROS/NLRP3/GSDMD-mediated pyroptosis pathway, thereby reducing cartilage degradation and inflammatory cytokine release.^36^ Ebata et al. demonstrated that inflammatory macrophage-derived extracellular vesicles exacerbated osteoarthritis by inducing chondrocyte pyroptosis, suggesting this pathway as a promising therapeutic target.^32^ Specifically, NLRP3 serves as the core component of the inflammasome.^38,39^ Its activation is significantly controlled by post-translational modifications of phosphorylation and dephosphorylation.^40–51^

In this study, we discovered that ERK5 regulated the cartilage pyroptosis through bioinformatics analysis and in vitro validation. Inhibition of NLRP3 activation rescued constitutive ERK5 activation-induced osteoarthritis enhancement. Specially, Zhang et al. reported that ADP/P2Y1 promoted inflammatory bowel disease via ERK5-mediated NLRP3 inflammasome activation.^52^ Those results indicated a regulatory role for ERK5 in NLRP3 inflammasome. Further Co-IP revealed a direct interaction between ERK5 and NLRP3. Structural analysis revealed that ERK5 bound to the PYD region of NLRP3 and phosphorylated it at Ser198. This site is highly conserved across multiple species. Notably, Ser198 is the same residue previously reported to be phosphorylated by JNK1.^47^ Since both JNK1 and ERK5 belong to the MAPK family, Ser198 of NLRP3 emerges as a key site for regulating pyroptosis by the MAPK cascade.

Oroxylin A, a main active flavonoid component extracted from *Oroxylum indicum*. It has strong antioxidant and anti-inflammation activity.^53^ Previous studies have shown that Oroxylin A is effective in alleviating inflammation in multiple chronic diseases. Ma et al. reported that Oroxylin A ameliorated psoriasiform skin inflammation via directly targeting p62 and inhibiting M1 macrophage polarization.^54^ Additionally, Oroxylin A attenuated the abnormal subchondral bone remodeling in osteoarthritis progression.^55–57^ In our study, we identified that Oroxylin A had strong binding affinities to ERK5 at ATP-binding site to inhibit its activity by virtual screening and in vitro validation. Further animal experiment showed that Oroxylin A effectively ameliorated the progression of DMM and aging-induced OA in mice. To assess the pharmacokinetic and pharmacodynamic characteristics of Oroxylin A, we used the *SwissADME* tool to analyze its ADME properties. Results showed that it complied with the Lipinski class principle and had a favorable gastrointestinal absorption effect (Fig. S8). These advantages position Oroxylin A as a safe and effective candidate for OA treatment.

There are several limitations in our study. Our investigation focused exclusively on cartilage, rather than exploring the role of ERK5 in other joint tissues. Further research is warranted in this area to delineate the involvement of ERK5 in OA pathogenesis. The regulatory effects of constitutive ERK5 activation on chondrocytes were examined using an intra-articular AAV injection model, it can be further validated using genetically modified animals with constitutive ERK5 activation. Potential effects of Oroxylin A through other mechanisms were not investigated. Further research using larger animal models and subsequent clinical trials are essential to validate the effectiveness of Oroxylin A for treating human OA. Since only male mice were used in this study, potential sex differences in osteoarthritis development and ERK5 function cannot be excluded. Further investigation using both sexes is warranted to clarify this issue.

**Fig. 8:**
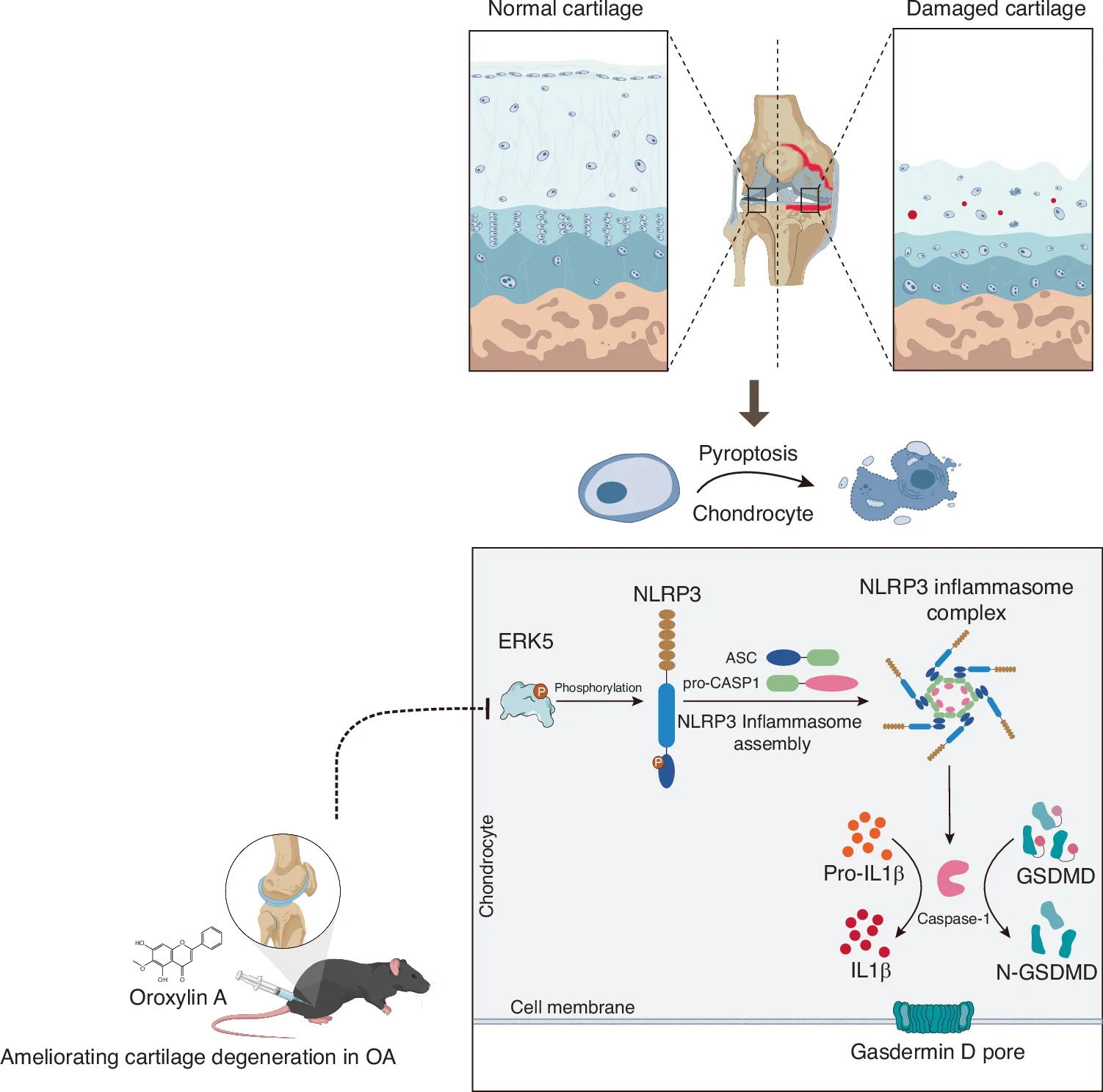
Graphic illustration of the function of ERK5 in OA progression. Under pathological conditions, ERK5 exacerbates osteoarthritis severity by driving NLRP3 inflammasome activation through NLRP3 phosphorylation. This process leads to IL-1β maturation and secretion, as well as the induction of pyroptosis. Specifically, Oroxylin A can inhibit the phosphorylation of ERK5 and ameliorate cartilage degeneration in OA

In summary, our study demonstrates that ERK5 plays a pivotal role in orchestrating NLRP3 inflammasome activation and subsequently inducing chondrocytes inflammation and pyroptosis. Genetic deletion of ERK5 in chondrocytes and its pharmacological inhibition both effectively attenuate cartilage degeneration in OA model (Fig. 8). Collectively, our findings provide convincing evidence that ERK5 is a promising therapeutic target for OA treatment.

## MATERIALS AND METHODS

### Human cartilage samples

Ethical approval for this study was granted by the Ethics Committee of the First Affiliated Hospital of Sun Yat-sen University (Approval No. 2023-177). Written informed consent was obtained from all participants prior to sample collection. Articular cartilage specimens were obtained from patients undergoing knee replacement surgery, with normal control cartilage obtained from trauma patients who had no history or evidence of osteoarthritis. Detailed patient characteristics are summarized in Table S1.

### Animals

All mice were maintained in the Laboratory Animal Center of Sun Yat-sen University under specific pathogen-free (SPF) conditions. All experimental protocols were approved by the Institutional Animal Care and Use Committee of Sun Yat-sen University (Approval No. SYSU-IACUC-2022-001665). *Erk5*^flox/flox^ mice were generated by Cyagen Biosciences as previously described.^26^ *Col2a1-CreER*^*T2*^ transgenic mice were obtained from The Jackson Laboratory. To generate *Col2a1-CreER*^*T2*^; *Erk5*^flox/flox^ mice (conditional knock out, CKO), *Erk5*^flox/flox^ mice were crossed with *Col2a1-CreER*^*T2*^ mice, yielding *Col2a1-CreER*^*T2*^; *Erk5*^flox/+^ offspring, which were subsequently bred with *Erk5*^flox/flox^ mice. The routine tail DNA genotyping was performed using the primers in Table S3. Mice were maintained in SPF conditions (≤ 5 per cage) at 22–26 °C with 40%–60% humidity, 12 h/12 h light-dark cycles, and were housed with ad libitum access to standard food and water. Mice were randomly allocated into different groups, with sample sizes as detailed in the figure legends. Mice were euthanized via CO₂ inhalation or isoflurane anesthesia (1.5%–2% in O₂) for the collection of knee specimens at indicated time, with subsequent histomorphometry and radiologic assessments.

### Isolation and culture of primary articular chondrocytes

Primary articular chondrocytes were isolated according to established protocols.^58^ In brief, knee cartilage tissues were dissected from postnatal day 5 (P5) mice under sterile conditions and minced into small fragments. The cartilage pieces were then digested overnight at 37 °C in 0.2% type II collagenase (Sigma-Aldrich). After digestion, the suspension was filtered and centrifuged at 100 × *g* for 5 min. The pelleted cells were cultured in DMEM/F12 (1:1) complete medium (Gibco) supplemented with 10% fetal bovine serum and 1% penicillin‑streptomycin, and maintained at 37 °C in a 5% CO₂ atmosphere. Cells at passage 1 were used for subsequent experiments.

### Micromass culture

The micromass culture was performed as demonstrated previously.^59^ For cell seeding, 15 μL droplets with 3 × 10^5^ cells were placed centrally in 24-well plate wells. After allowing 2 h for cell adhesion, 500 μL of chondrogenic medium was supplemented. Micromass for chondrogenesis was assessed by Alcian blue staining.

### CCK8-assays

The cell viability was evaluated with the Cell Counting Kit-8 (CCK-8) assay. Cells were seeded in 96-well plates at a density of 1.5 × 10^4^ cells per well. Upon reaching 75% confluence, the indicated treatments were applied. Next, 10 µL of CCK-8 reagent was added to each well, followed by incubation for 1 h. Absorbance at 450 nm was then recorded using a microplate reader (Tecan Sunrise).

### Real-time quantitative PCR

Total RNA was isolated with TRIZOL reagent (Invitrogen). RNA concentration and purity were evaluated on a Nanodrop 2000 spectrophotometer (Thermo Scientific). Reverse transcription was carried out with 1 000 ng of total RNA according to the protocol of the NovoScript® 1st Strand cDNA Synthesis Kit (Novoprotein). Quantitative PCR was subsequently performed on a Roche LightCycler 96 system using NovoStart® SYBR qPCR SuperMix Plus (Novoprotein). Relative mRNA expression was calculated via the 2^−ΔΔCt^ method, normalizing to β‑Actin as the housekeeping gene. Primer sequences are listed in Table S4.

### Supernatant protein extraction

Protein was precipitated from collected cell culture supernatants. After centrifugation to remove debris, one‑fourth volume of chloroform and one volume of methanol were added to the cleared supernatant. Samples were mixed thoroughly and centrifuged at 12 000 r/min for 5 min at room temperature. The upper methanol phase was carefully discarded. Two volumes of fresh methanol were then added to the remaining interphase and pellet, followed by a second centrifugation under identical conditions. The resulting pellet was air‑dried at room temperature or briefly dried on a heating block at 50 °C for 5 min. Dried protein was finally resuspended in an appropriate cell lysis buffer.

### Western blot analysis

Total protein was isolated using ice-cold RIPA lysis buffer (Cell Signaling Technology) containing a protease inhibitor cocktail, with incubation on ice for 30 min. Lysates were clarified by centrifugation at 12 000 × *g* for 20 min at 4 °C. Protein concentration was determined with a BCA Protein Assay Kit. After denaturation at 100 °C for 10 min, equal amounts of protein were resolved on 10% SDS‑PAGE gels and electrotransferred onto PVDF membranes (Millipore). Membranes were blocked in 5% non‑fat milk for 1 h at room temperature, followed by overnight incubation at 4 °C with primary antibodies (see Table S2). After washing, membranes were incubated with HRP‑conjugated secondary antibodies (Jackson) for 1 h at room temperature. Signal detection was performed using an enhanced chemiluminescence (ECL) system (Millipore).

### Co-immunoprecipitation assay

Whole-cell lysates were generated utilizing RIPA lysis buffer. Following centrifugation at 12 000 × *g* for 10 min at 4 °C, the supernatant was collected and incubated with primary antibodies at 4 °C with rotation for 12 h. Then, 40 μL of 50% Protein A + G Sepharose beads (Santa Cruz Biotechnology) were added, followed by incubation at 4 °C with rotation for 2 h. The beads were washed three times with lysis buffer, 15 min each time. Finally, the supernatant was removed, and SDS-PAGE loading buffer was added to elute the precipitated proteins from the beads by boiling at 70 °C for 10 min. The proteins were analyzed by immunoblotting using specified antibodies.

### Histological analysis and immunohistochemistry (IHC)

Mouse knee joints underwent fixation in 4% paraformaldehyde (PFA) for 48 h, followed by decalcification in 10% EDTA (pH 7.4) over a 30-day period. After dehydration, specimens were embedded in paraffin and sectioned at 5 μm thickness. Sections were dewaxed, rehydrated, and then stained using a modified Safranin O fast green staining kit (Solarbio Life Sciences, Beijing, China) and Alcian blue staining. Cartilage degradation was assessed according to the Osteoarthritis Research Society International (OARSI) scoring criteria. For IHC, tissue sections were heated at 65 °C overnight for antigen retrieval. Endogenous peroxidase activity was suppressed by incubation in 3% hydrogen peroxide for 10 min, followed by blocking with 10% goat serum for 30 min. Primary antibodies (detailed in Table S2) were applied and incubated overnight at 4 °C. Subsequently, sections were treated with horseradish peroxidase (HRP)-conjugated secondary antibodies, and immunolabeling was developed using a 3,3′-diaminobenzidine (DAB) substrate system. Imaging was conducted with a Leica DM14000B microscope (Wetzlar, Germany).

### Immunofluorescence (IF)

Following dewaxing and rehydration, tissue sections were subjected to antigen retrieval by overnight incubation in retrieval buffer at 65 °C. Sections were then blocked using 10% goat serum for 30 min. For cell samples, coverslips were fixed in 4% paraformaldehyde for 15 min, washed three times with phosphate-buffered saline (PBS), and blocked as previously. Both tissue sections and cell coverslips were incubated with primary antibodies (specified in Table S2) overnight at 4 °C. Afterwards, samples were treated for 1 h at room temperature with Alexa Fluor 488- or Alexa Fluor 594-conjugated secondary antibodies (Invitrogen; dilution 1:600). Visualization and image acquisition were performed at appropriate excitation wavelengths using an Olympus BX63 fluorescence microscope (Tokyo, Japan).

### Edu assay

Cell proliferation was evaluated using the E-Click EdU Cell Proliferation Imaging Assay Kit (Green, Elab Fluor® 488) in accordance with the manufacturer’s guidelines.^60^ Briefly, cells in each well were initially incubated with EdU solution (50 μmol/L per well) for 2 h. Cells were fixed with 4% paraformaldehyde for 15 min and then permeabilized using 0.5% Triton X-100 for 20 min. The cells were subsequently treated with Click reaction solution for 30 min to allow fluorescent labeling of EdU-incorporated DNA. After washing with phosphate-buffered saline (PBS) for 10 min, nuclei were counterstained with DAPI for 10 min at room temperature.

### ELISA

Cellular supernatant levels of IL-1β and Caspase-1 were quantified following the manufacturer’s instructions using enzyme-linked immunosorbent assay (ELISA) kits. Absorbance was measured at 450 nm on a Tecan Sunrise microplate reader.

### In vitro kinase assay

Recombinant FLAG-NLRP3 or FLAG-NLRP3-S198A was employed as the substrate and incubated with recombinant ERK5 and CA-MEK5 in a kinase assay buffer composed of 12.5 mmol/L β-glycerophosphate, 0.25 mmol/L DTT, 25 mmol/L MgCl_2_, 2 mmol/L EDTA, 25 mmol/L MOPS (pH 7.2) and 5 mmol/L EGTA, and at 40 °C for 45 min. The reactions were terminated by adding SDS-PAGE sample buffer.

### OA animal models

OA was induced in 10-week-old male C57BL/6 mice via surgical destabilization of the medial meniscus (DMM). Following anesthesia induced by intraperitoneal injection of 50 g/L chloral hydrate, a longitudinal incision was performed on the medial aspect of the right knee joint to expose the medial meniscotibial ligament (MMTL). The ligament was transected, and the surgical site was irrigated with sterile saline before closure with layered sutures. Postoperative amoxicillin was administered to minimize the risk of infection.

### Intra-articular administration

Intra-articular delivery of adeno-associated virus (AAV), The pAAV-CMV-MCS-HA-P2A-eGFP (AAV-NC), pAAV-CMV-*Mek5*(S313D; T315D)-HA-P2A-eGFP (AAV-CA-*Mek5*), pAAV-CMV-*Erk5*-HA-P2A-eGFP (AAV-*Erk5*-WT), pAAV-CMV-*Erk5*(T218A; Y220F)-HA-P2A-eGFP (AAV-*Erk5*-MUT) were constructed by GeneChem (Shanghai, China). The titers of each virus are as follows: AAV-*Erk5*-WT (2 × 10^10^ vg/mL) and AAV-*Erk5*-MUT (1.5 × 10^11^ vg/mL). Different viruses were mixed prior to injection: *Erk5*OE-WT group (5 μL AAV-CA-*Mek5* + 5 μL AAV-*Erk5*-WT) and *Erk5*OE-MUT group (5 μL AAV-CA-*Mek5* + 5 μL AAV-*Erk5*-MUT). A total of 10 μL virus mixture was administered intra-articularly into the right knee joints of mice 2 weeks post-surgery, using a Hamilton microsyringe. Control group was treated with AAV-NC for the same periods. Immunofluorescence analysis was performed to detect GFP expression, confirming effective AAV transduction (Fig. S4A). Oroxylin A was delivered via weekly intra-articular injection at different doses indicated in the figure legends.

### Open field test

The open field test was used to assess spontaneous locomotor activity in mice. Each mouse was tested once before DMM modeling and then weekly. Mice were acclimatized to the testing environment for 30 min before the experiment. Each mouse was placed in a 1 m × 1 m × 5 m open-field chamber, and a digital camera (mounted 2 m above) recorded its movement for 2 min. After each trial, the chamber was cleaned with alcohol before testing the next mouse. All movement trajectories were analyzed to calculate the total distance traveled.

### Von Frey test

Animals were allowed to acclimate for 30 min in clear plexiglass enclosures positioned atop a elevated wire grid platform. Mechanical sensitivity was evaluated using a series of von Frey filaments (0.07–2 g; Stoelting) applied in ascending order to the plantar surface of the hind paw. Each filament was pressed until bending, with a 5–10 s interval between applications. Every filament was applied 10 times per paw, and the number of withdrawal responses was documented. The paw withdrawal threshold was determined as the lowest filament force that elicited a withdrawal response in at least five out of ten trials (≥ 50% response rate).

### Micro-computed tomography (CT) analysis

The progression of osteophytes and alterations in the subchondral bone of knee joints were assessed via Micro-CT scanning, following previously established protocols.^60^ Briefly, the intact knee joints obtained from OA model mice were scanned with a micro‑CT system (SkyScan1276, Bruker) at a resolution of 10 µm and scanning parameters of 70 kV/200 µA. Image reconstruction was performed using NRecon v1.6. Subsequent histomorphometric analysis was carried out with CTAn v1.17 on a defined region of interest (ROI) within the tibial subchondral bone to quantify bone volume per tissue volume (BV/TV), trabecular number (Tb.N), and trabecular separation (Tb.Sp). Three‑dimensional visualization was achieved by generating models with Mimics Research software (v21.0).

### RNA-sequencing

Total RNA was isolated from TNF-α-treated primary articular chondrocytes derived from *Erk5*^flox/flox^ mice following transfection with Ad-GFP or Ad-Cre. The RNA‑sequencing procedure, conducted by Novogene Biotechnology (Beijing, China), included three biological replicates per group. A cDNA library was prepared and sequenced according to the standard Illumina protocol. Gene expression levels were quantified with featureCounts (v2.0.1), and differential expression analysis was performed using the DESeq2 R package (v1.20.0). *P*‑values were adjusted via the Benjamini–Hochberg method to control the false discovery rate (FDR). Differentially-expressed genes (DEGs) were regarded as statistically significant with |log_2_fold change| > 1 and *P*-value < 0.05.

### Molecular docking

The procedure for molecular docking followed previously established methods.^60^ The three-dimensional (3D) structure of ERK5 was acquired from the Protein Data Bank (PDB code: 4ZSG). Virtual screening of a natural small molecule compound library (Target Mol, Boston, USA) against ERK5 protein was performed using AutoDock Vina and AutoDock Tools 1.5.6. A few top-ranked compounds from Target Molecule Corp were selected for further testing.

### Molecular dynamics simulation

The MD simulations were conducted using Gromacs2020.6.^61,62^ The ARMM36 force field was employed to define topologies for the protein and ligand. The system was placed within a cubic TIP3P water box (10 Å edge length) and neutralized by adding Na^+^/Cl^−^ ions. Prior to production runs, energy minimization (5 000 steps, steepest descent algorithm) was conducted to eliminate steric clashes. Subsequently, the system underwent NVT and NPT equilibration following standard protocols. A 30-ns production MD simulation was carried out, and trajectory analysis was performed using Gromacs utilities.

### Statistical analysis

Statistical analysis was performed using GraphPad Prism 8. For comparing multiple groups, one-way analysis of variance (ANOVA) with Tukey’s multiple comparisons test was applied (parametric test). Nonparametric data (e.g., OARSI scores) were analyzed using the Kruskal–Wallis test with multiple comparisons. When comparing only two groups, Student’s paired or unpaired *t*-test (parametric or nonparametric) was used to detect significant treatment effects. *P*-value < 0.05 was considered statistically significant. All data are presented as mean ± SEM.

## Supplementary information

Supplementary Document

## Data Availability

Data are available upon reasonable request. The data that support the findings in this study are available from the corresponding author upon request.
