# Supplementary material for: Inhibition of ERK5 activity ameliorates osteoarthritis by suppressing NLRP3 inflammasome-mediated chondrocyte pyroptosis
Source: Bone Res. 2026 Sep 2;14:95. doi: 10.1038/s41413-026-00573-x (PMC13538362; doi:10.1038/s41413-026-00573-x)
Supplement: Supplementary file 1 [file 41413_2026_573_MOESM1_ESM.docx]

**Supporting Information**

**Inhibition of ERK5 Activity Ameliorates Osteoarthritis by Suppressing NLRP3 Inflammasome-mediated Chondrocyte Pyroptosis**

*Baolin Zhang ^a b #^, Peihang Fang ^a b #^, Jiahui Long ^c #^, Deying Su ^c^, Hengyu Liu ^a b^, Ricong Weng ^a b^, Gengjia Chen ^a b^, Yingtong Chen ^a b^, GuanHong Chen ^a b^, Zhiheng Liao* *^a b^, Taifeng Zhou ^a b^, Shuqing Chen ^d *^, Peiqiang Su ^a b *^, Caixia Xu^a b c *^*

^a^ *Department of Spine Surgery, the First Affiliated Hospital of Sun Yat-sen University, Guangzhou 510080, China*

^b^ *Guangdong Provincial Key Laboratory of Orthopedics and Traumatology, Department of Spine Surgery, the First Affiliated Hospital of Sun Yat-sen University, Guangzhou 510080, China*

^c^ *Research Center for Translational Medicine, First Affiliated Hospital, Sun Yat-sen University, Guangzhou 510006, China*

*^d^ Department of Traditional Chinese Medicine, the First Affiliated Hospital, Sun Yat-sen University, Guangzhou 510006, China*

**# These authors contributed equally to this work.**

**Corresponding authors**

Shuqing Chen^*^

*Department of Traditional Chinese Medicine, the First Affiliated Hospital, Sun Yat-sen University, Guangzhou 510006, China. chshuq@mail.sysu.edu.cn*

Peiqiang Su^*^

*^*^* *Department of Orthopaedic Surgery, First Affiliated Hospital, Sun Yat-sen University, Guangzhou, Guangdong, China.* [*supq@mail.sysu.edu.cn*](mailto:supq@mail.sysu.edu.cn)

Caixia Xu^*^

*^*^* *Research Center for Translational Medicine, First Affiliated Hospital, Sun Yat-sen University, Guangzhou 510006, China.* [*xucx3@mail.sysu.edu.cn*](mailto:xucx3@mail.sysu.edu.cn)

**Fig. S1. ERK5 knockdown inhibits the extracellular matrix (ECM) degradation of primary mouse chondrocytes under inflammation condition in vitro.** (A) Immunoblotting of ERK5 in primary mouse chondrocytes following knocking down *Erk5*. (B) Immunoblotting of relevant proteins in primary articular chondrocytes following transfection with si-ERK5 or si-NC and then treated with or without TNF-α (20 ng/mL) for 48 h. One-way ANOVA followed by Tukey’s multiple comparisons is used for analysis. The bar graph shows the quantification of the relative protein (mean±SD, *p < 0.05, **p < 0.01).

**Fig. S2. Cartilage-specific *Erk5* knockout mice were constructed via Cre-loxP system.** (A) Schematic diagram of gene targeting design. (B) PCR amplification confirming the mouse genotyping results. (C) Protein and mRNA levels of ERK5 in primary articular chondrocytes isolated from CKO mice, assessed by western blotting and qRT-PCR following treatment with 4-hydroxytamoxifen (4-OHT, 1 mM). P-values were calculated using two-tailed unpaired *t* test. (D) Immunohistochemical staining of ERK5 in knee joint sections from 10-week-old WT and CKO littermates after tamoxifen (TMX) induction at 8 weeks of age. Data are expressed as mean ± SD; ***p < 0.001. Scale bar: 100 μm.

**Fig. S3. ERK5 knockout alleviates mice pain during OA progression.** (A) Open field movement experiment results of WT and CKO mice at various time points following DMM surgery. (B) Mechanical allodynia evaluated using the Von Frey test in WT and CKO mice at indicated weeks post-DMM surgery. Two-way ANOVA test is used for analysis. Data are presented as mean ± SD.

**Fig. S4.** **Signals examination in mice knee cartilage after AAV injection.** (A) Detecting the expression of GFP 1 month after injection of AAV. Scale bar, 100 μm. (B) Quantification of the p-ERK5/total ERK5 ratio by immunofluorescence in all groups. Scale bar, 100 μm. One-way ANOVA followed by Tukey’s multiple comparisons is used for analysis. Data are expressed as mean ± SD; *p < 0.05.

**Fig. S5. Oroxylin A enhances chondrocyte viability in an inflammatory environment.** (A) PCR analysis of *Erk5* expression in chondrocytes following treatment with various compounds. One-way ANOVA is used for analysis. (B) Protein levels of ERK5 and p-ERK5 across different treatment groups. (C) Examination of phosphorylated NLRP3 levels in chondrocytes with or without Oroxylin A treatment under TNF-α stimulation. (D) Immunoblotting of indicated proteins in different groups. (E) Co‐IP analysis of ERK5-NLRP3 interaction in chondrocytes. (F) Western blot analysis of related proteins in chondrocytes with or without ERK5 activation. (G) Cell proliferation assessed by EdU staining in each experimental group. Two-way ANOVA test is used for analysis. Data are shown as mean ± SD; # p < 0.05 compared to the control group; ns (not significant), *p < 0.05, **p < 0.01 compared to the TNF‑α treatment group. Scale bar: 100 μm

**Fig. S6. Oroxylin A suppresses chondrocyte degeneration induced by inflammation.**(A) Immunoblotting analysis of COL2A1, ADAMTS5, MMP13 and β-Actin in different groups. (B) Alcian blue staining were shown for chondrocyte micromass cultures treated for 5 days with TNF-α, Oroxylin A (10 and 20 μM), or celecoxib (10 μM). (C) and (D) COL2A1 and MMP13 expression was measured via immunofluorescence staining. Scale bars, 100 μm. Two-way ANOVA test is used for analysis. Data are presented as the mean ± SD; ns (non-significant), *p < 0.05, **p < 0.01, ***p < 0.001.

**Fig. S7. Oroxylin A ameliorates OA-associated pain behaviour.** (A) Open field movement experiment results of different mice at various post-surgical weeks following DMM surgery. (B) Quantitative analysis of results from (A). (C) Von Frey testing results of different mice at various post-surgical weeks following DMM surgery. Two-way ANOVA with Bonferroni post hoc test was used. Data are presented as the mean ± SD; * Oroxylin A (2mg/kg) vs DMM, # Oroxylin A (4mg/kg) vs DMM, ^ Celecoxib vs DMM. ns (non-significant), *, #, and ^ p < 0.05; **, ##, and ^^ p < 0.01;

**Fig. S8. The analysis of ADME properties of Oroxylin A via *SwissADME* tool.**

**Table S1. Clinical features of patients**

| **Patient Number** | **Age** | **Gender** | **Diagnosis** |
| --- | --- | --- | --- |
| #1 | 83 | Female | Osteoarthritis (right knee) |
| #2 | 75 | Female | Osteoarthritis (right knee) |
| #3 | 80 | Male | Osteoarthritis (right knee) |
| #4 | 78 | Female | Osteoarthritis (left knee) |
| #5 | 82 | Female | Osteoarthritis (right knee) |
| #6 | 65 | Male | Osteoarthritis (left knee) |
| #7 | 72 | Female | Osteoarthritis (right knee) |
| #8 | 66 | Female | Osteoarthritis (right knee) |
| #9 | 70 | Male | Osteoarthritis (left knee) |
| #10 | 68 | Male | Osteoarthritis (right knee) |
| #11 | 79 | Female | Osteoarthritis (right knee) |
| #12 | 82 | Female | Osteoarthritis (right knee) |
| #13 | 27 | Male | Fracture (right femoral) |
| #14 | 15 | Female | Fracture (right femoral) |
| #15 | 45 | Male | Fracture (left femoral) |
| #16 | 37 | Male | Fracture (right femoral) |
| #17 | 32 | Male | Fracture (right femoral) |
| #18 | 46 | Female | Fracture (left femoral) |
| #19 | 35 | Male | Fracture (left femoral) |

**Table S2: Lists of antibodies.**

| **Antibody** | **SOURCE** | | **IDENTIFIER** | **DILUTION** |
| --- | --- | --- | --- | --- |
| COL2A1 | Abcam | Cat# ab34712 | | WB (1:1000)  IF (1:200) |
| p-ERK5^T218/Y220^ | CST | Cat# 3371 | | WB (1:1000)  IHC (1:200) |
| ERK5 | CST | Cat# 3372 | | WB (1:1000)  IHC (1:200) |
| MMP13 | Abcam | Cat# ab39012 | | WB (1:1000)  IHC (1:200)  IF (1:200) |
| ADAMTS5 | Abcam | Cat# ab41037 | | WB (1:1000)  IHC (1:200) |
| β-Actin | Affinity | Cat# T0022 | | WB (1:10000) |
| Aggrecan | Abcam | Cat# ab3778 | | IHC (1:200) |
| Caspase-1 | Proteintech | Cat# 22915-1-AP | | WB (1:1000)  IHC (1:200) |
| IL-1β | CST | Cat# 12242 | | WB (1:1000)  IHC (1:200) |
| NLRP3 | AdipoGen | Cat# AG-20B-0014 | | WB (1:1000)  IHC (1:200) |
| ASC | Abiowell | Cat# AWA58244 | | WB (1:1000) |
| GSDMD | Proteintech | Cat# 20770-1-AP | | WB (1:1000) |
| Phosphoserine | Abcam | Cat# ab9332 | | WB (1:1000) |

**Table S3: Primers for genotyping.**

| **Gene** | **Direction** | **Sequence (5’-3’)** |
| --- | --- | --- |
| *Erk5^fl/fl^* | Forward | TCCTTACAGCCAGGCAGACTCAGC |
|  | Reversed | ACTCAAGCCAAGCCTCACCTGCCA |
| *Col2a1-CreER^T2^* | Forward | ATCCGAAAAGAAAACGTTGA |
|  | Reversed | ATCCAGGTTACGGATATAGT |

**Table S4: Primers for qPCR.**

| **Gene** | **Direction** | **Sequence (5’-3’)** |
| --- | --- | --- |
| *Actb* | Forward | CATTGCTGACAGGATGCAGAAGG |
|  | Reversed | TGCTGGAAGGTGGACAGTGAGG |
| *Col2a1* | Forward | GCTGGTGAAGAAGGCAAACGAG |
|  | Reversed | CCATCTTGACCTGGGAATCCAC |
| *Adamts5* | Forward | CTGCCTTCAAGGCAAATGTGTGG |
|  | Reversed | CAATGGCGGTAGGCAAACTGCA |
| *Mmp13* | Forward | TGTTTGCAGAGCACTACTTGAA |
|  | Reversed | CAGTCACCTCTAAGCCAAAGAAA |
| *Acan* | Forward | CAGGCTATGAGCAGTGTGATGC |
|  | Reversed | GCTGCTGTCTTTGTCACCCACA |
| *Erk5* | Forward | TAAACCCTCTAACCTTCTGGTCA |
|  | Reversed | GAGGTCGATTGCCTGCGTA |

**Western Blot**
